# A Rare and Treatable Cause of Medullar Claudication: Spinal Dural Arteriovenous Fistula

**DOI:** 10.31486/toj.18.0026

**Published:** 2019

**Authors:** Celine Derollez, Celine Tard, Apolline Kazémi, Patrick Vermersch, Jean-Pierre Pruvo

**Affiliations:** ^1^ Department of Neurology, Roger Salengro Hospital, Lille University Medical Center, Lille Cedex, France; ^2^ Degenerative and Vascular Cognitive Disorders, U1171, University Lille, INSERM, Centre Hospitalier Universitaire de Lille, Lille, France; ^3^ Department of Neuroradiology, Roger Salengro Hospital, Lille University Hospital Center, Lille Cedex, France; ^4^ University Lille, INSERM, Centre Hospitalier Universitaire de Lille, Lille, France

**Keywords:** *Angiography*, *central nervous system vascular malformations*, *dural arteriovenous fistula*, *embolization–therapeutic*, *intermittent claudication*, *spinal cord diseases*

## Abstract

**Background:** Spinal dural arteriovenous fistula is a rare and underdiagnosed disorder. Because of the nonspecific clinical presentation of the condition, patients are often referred to different specialists, resulting in delayed diagnosis.

**Case Report:** A 76-year-old male presented with a 1-month history of gait trouble. His impairment was asymmetric, distally predominant, sensitive, and motor. Symptoms worsened with standing and walking. The patient also had sphincterial dysfunction. Classic spinal cord magnetic resonance imaging (MRI) showed an extended hypersignal indicating nonspecific myelopathy. Repeat spinal cord MRI that included a T2 spin echo sequence revealed abnormalities suggesting dural arteriovenous fistula. Medullar angiography confirmed the diagnosis, and endovascular treatment was successfully performed. Six months posttreatment, the patient reported resolution of his neurologic disabilities except for some residual paresthesia in his inferior limbs.

**Conclusion:** Physicians should be aware of the specific abnormalities shown on spinal cord MRI that indicate dural arteriovenous fistula, as well as the criteria for performing medullar angiography, so that the condition can be diagnosed and treated in a timely manner. Early therapeutic treatment is the principal prognosis factor.

## INTRODUCTION

Spinal dural arteriovenous fistula is a rare and underdiagnosed disorder, although the condition has a characteristic neuroradiologic presentation that can indicate the diagnosis. We present the case of a patient with a spinal dural arteriovenous fistula that highlights the importance of the neuroradiologist's experience and collaboration with the clinician for a rapid and correct diagnosis that resulted in timely interventional treatment.

## CASE REPORT

A 76-year-old male presented with a 1-month history of gait trouble. His medical history was unremarkable except for hypertension and a recent surgery for left knee prosthesis. He described his impairment as an asymmetric and distally predominant deficit that limited his walking perimeter and his ability to stand for prolonged periods. He had distal sensory disturbances such as paresthesia (swarming), dysesthesia, and allodynia when he walked or stood for long periods. He reported numbness in the soles of his feet and right-sided low back pain with no sciatica radicular path. The patient's symptoms had worsened gradually. He had no weight loss, infection, fever, or extraneurologic symptoms.

Neurologic examination confirmed the patient's complaints with findings of protopathic hypoesthesia, tactile allodynia, numbness in the soles of his feet, and hypopallesthesia in the inferior limbs, especially on the right side. The patient had a predominantly distal motor deficit and a 50 m walking perimeter. Reflexes were normal. He did not complain about sphincterial dysfunction, but analysis revealed residual urine of 400 cc.

**Figure 1.**
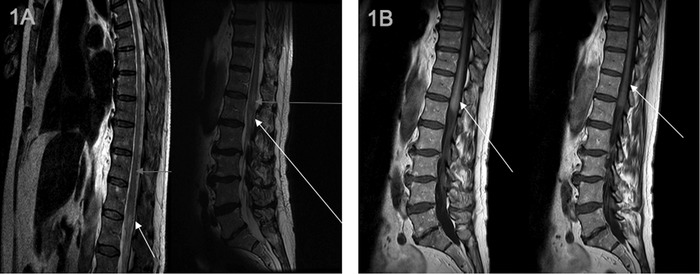
**Sagittal magnetic resonance imaging with (A) T2 spin echo (TSE) sequence reveals hypersignal of the spinal cord from T6 to the terminal cone (arrows) and flow voids behind the spinal cord that are (B) enhanced with gadolinium injection. The hypersignal of the spinal cord on TSE sequence corresponds to chronic hypoxic congestive myelopathy attributable to venous hyperpressure hindering venous return and caused by an arteriovenous shunt in spinal cord vascularization. The arrows in views A and B correspond to congested small venous vessels that are hard to visualize because they are quite thin.**

Symptoms, walking complaints, and the patient's age suggested a narrow lumbar canal. Routine medullary spinal cord magnetic resonance imaging (MRI) scan to study bones, medullar discs, and nervous vascular conflict revealed a nonspecific medullar extended hypersignal. Laboratory tests for inflammation disorders were negative.

The results suggested a vascular etiology. A second spinal cord MRI including a specific T2 spin echo sequence showed hypersignal of the spinal cord from T6 to the terminal cone that was associated with abnormal flow voids behind the spinal cord (Figure 1A). Flow voids were enhanced after an injection of gadolinium (Figure 1B). MRI findings suggested a spinal dural intravenous fistula.

**Figure 2.**
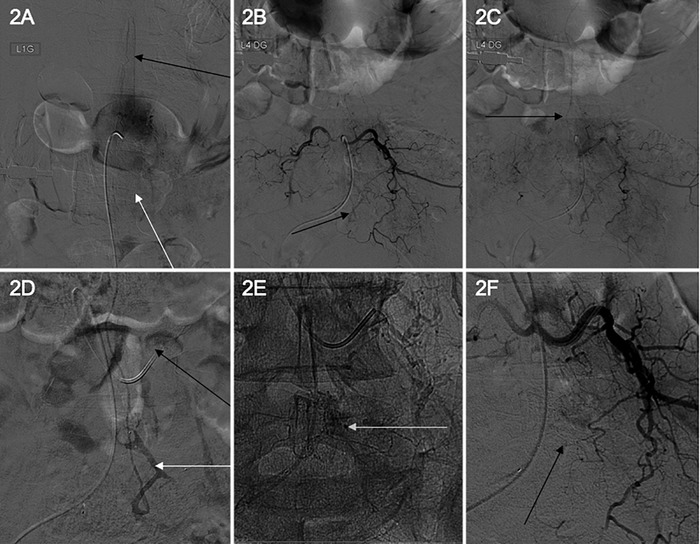
**Images from the (A) first and (B to F) second medullar angiographies performed on the patient. During the first angiography, the end of the anterior artery injection was visible (A, dark arrow), but the venous return of the spinal cord could not be visualized because it was slowed by a dural arteriovenous fistula (A, white arrow). During the second angiography, an uncommon vessel on T4 left level was visualized (B), draining in the spinal vein (C) and corresponding to the dural arteriovenous fistula. Endovascular treatment was performed by catheterization (D, black arrow) of the fistula point (D, white arrow). Injection of the embolic agent (E, white arrow) resulted in the disappearance of the dural arteriovenous fistula (F, black arrow).**

Technical difficulties associated with aortic atheroma linked to the patient's age prevented completion of medullar angiography under general anesthesia. General anesthesia is necessary because of required episodes of apnea. As a result, no significant abnormalities were detected (Figure 2A). A second medullar angiography performed by an experienced neuroradiologist allowed definite diagnosis of a left T4-level spinal dural arteriovenous fistula (Figures 2B and 2C). In the absence of anesthetic, vascular anatomic, and technical contraindications, endovascular treatment by embolization of the fistula point was successfully performed during the angiography (Figures 2D, 2E, and 2F). Six months after treatment, the patient reported complete recovery of his motor and sphincterial dysfunctions. The only remaining symptom was paresthesia of his inferior limbs.

## DISCUSSION

Although spinal dural arteriovenous fistulas account for 70% to 80% of spinal vascular malformations, they are still rare and underdiagnosed.^1^ Spinal dural arteriovenous fistulas principally affect middle-aged men^2^ and correspond to the presence of an arteriovenous shunt located in the spinal dura matter between a dural artery and a radicular vein. Arterialization of the spinal vein results in venous hyperpressure that hinders the venous return to the spinal cord and thus causes a chronic hypoxic congestive myelopathy,^1^ explaining why the clinical presentation is progressive myelopathy. Motor, sensitive, and genitourinary symptoms rapidly become worse. Symptoms depend on the level of dural arteriovenous fistula, begin distally, and progress upwardly to the level of the fistula.

The diagnosis is difficult to establish, and the mean delay to diagnosis is approximately 15 months.^2^ Because spinal dural arteriovenous fistula is not a well-known condition and has a nonspecific clinical presentation, patients may be referred to different specialists. Medullar claudication that affects middle-aged men suggests lumbar spinal stenosis requiring neurosurgical treatment (laminectomy). An MRI hypersignal in T2 sequence can nonspecifically be labelled as myelitis, referring to unspecific medullar inflammation with several possible etiologies (autoimmune, infectious, vascular) that require different types of therapeutic management.

Imaging is crucial to confirm the diagnosis of spinal dural arteriovenous fistula.^3^ Typically, the spinal cord MRI shows an unspecific T2 sequence hypersignal of the spinal cord that extends over more than 2 vertebrae and involves the terminal cone in 80% of cases.^2^ This T2 sequence hypersignal corresponds to chronic hypoxic myelopathy. Only with a T2 spin echo sequence can an abnormal perimedullar flow void, enhanced after an injection of gadolinium, be observed. The abnormal perimedullar flow void corresponds to a venous vascular dilatation attributable to a dural arteriovenous fistula. The level of the abnormality does not indicate the location of the dural arteriovenous fistula.

Medullar angiography is the gold standard for diagnosis and must be performed according to specific criteria. Medullar angiography must be strict anteroposterior view for characterization of the spinal anterior artery. Because of the thinness of the vessels, periods of apnea are required to visualize them, so patients should be under general anesthesia. Moreover, each radicular artery, supraaortic vessel, and internal iliac artery should be systematically catheterized for injection.^1^ Further, the injection must be long enough to avoid missing the dural arteriovenous fistula because it has a slow flow rate.

Once the fistula is visualized, a therapeutic discussion is necessary. Endovascular treatment can be performed by using a liquid agent (glue) to embolize the fistula point.^4^ In cases of contraindication or failure of embolization, surgical treatment should be discussed and performed as quickly as possible by using a clip or by coagulating the vein implicated in the arteriovenous shunt. The success rate of surgical treatment is higher than that of embolization (98% vs 46%).^5^

After treatment, symptoms generally improve, particularly motor symptoms. Prognosis depends on the level of severity of neurologic disturbance, early diagnosis, and early therapeutic treatment. A secondary worsening of symptoms can occur in cases of fistula recurrence, second fistula, or medullar venous insufficiency attributable to vein thrombosis resulting from sudden reduction of flow in the spinal vein system after treatment.^2^ Consequently, patients should be clinically monitored and undergo complete medullary angiography 6 months after treatment.

## CONCLUSION

Although dural arteriovenous fistula is the most common spinal vascular malformation, it is still underdiagnosed because of its nonspecific clinical presentation and many physicians’ lack of knowledge about its specific abnormalities shown on spinal cord MRI. Medullar angiography is the gold standard for diagnosis but must be performed according to strict criteria to identify the abnormality. Early therapeutic treatment is the principal factor affecting prognosis.

## Notes

This article meets the Accreditation Council for Graduate Medical Education and the American Board of Medical Specialties Maintenance of Certification competencies for Patient Care and Medical Knowledge.
